# Captain or deckhand? The impact of self-leadership on employees’ work role performance under remote work

**DOI:** 10.3389/fpsyg.2022.988105

**Published:** 2022-11-25

**Authors:** Ceyda Maden-Eyiusta, Suzan Ece Alparslan

**Affiliations:** ^1^Department of Entrepreneurship, Özyeğin University, Istanbul, Turkey; ^2^Department of Management, Kadir Has University, Istanbul, Turkey

**Keywords:** self-leadership, psychological empowerment, work role performance, supervisor close monitoring, remote work

## Abstract

Relying on self-determination theory, this study investigates the mediating role of psychological empowerment in the relationship between self-leadership and work role performance (task proficiency, task adaptivity, and task proactivity) in remote work settings. It also explores whether and how supervisor close monitoring moderates the indirect impact of self-leadership on work role performance. Hypotheses were tested using a two-study design including white-collar employees from a broad range of jobs and companies (Study 1) and employee-supervisor dyads working in small and medium-sized firms (Study 2) in Turkey. In Study 1, results showed that self-leadership had a positive indirect effect on employees’ work role performance through psychological empowerment. In Study 2, the cross-lagged two-wave design provided support for this indirect effect while demonstrating partial support for the moderating role of supervisor close monitoring. The current study contributes to research on self-leadership and work role performance by providing a detailed understanding of the motivational process through which self-leadership leads to increased work role performance. It also offers practical insights for enhancing self-leaders’ work role performance, particularly within the remote work context.

*“Self-leadership… is about influencing ourselves, creating the self-motivation and self-direction we need to accomplish what we want to accomplish.” [Charles C. Manz]*

## Introduction

The unexpected outbreak of the COVID-19 pandemic in 2020 and its rapid spread around the world have brought radical changes to work life, dramatically impacting the workplaces across the globe (Kniffin et al., 2021; Wang et al., 2021). Of these numerous changes, perhaps the most remarkable was the widespread, almost overnight switch to mandatory and full-time remote work, a transition which has created thoroughgoing challenges for employees as well as organizations (Chong et al., 2020; Costantini and Weintraub, 2022). In this new and unfamiliar context, employees have found themselves applying different behavioral and cognitive self-management strategies to cope with the challenges associated with remote work. Historically preceding these COVID-specific effects, developments in communication technology has also transformed the workplace providing employees with increased autonomy and discretion to monitor and control their own behavior (Gephart, 2002). Organizations and managers, on the other hand, adopt different techniques to monitor employees’ work while working in distant places (Wang et al., 2021). Taken together, these changes accentuate the importance of employees’ ability to lead themselves in the new work context by employing a particular set of self-leadership strategies.

Self-leadership refers to “a comprehensive self-influence perspective that concerns leading oneself toward performance of naturally motivating tasks as well as managing oneself to do work that must be done but is not naturally motivating” (Manz, 1986, p. 589). The concept first arose in 1980s (e.g., Manz, 1986) as an extension of self-management (e.g., Manz and Sims, 1980) and has attracted significant attention since then, as documented in several empirical and practitioner-oriented articles (e.g., Manz, 1992; Neck and Houghton, 2006; Marques-Quinteiro and Curral, 2012). Regarding its performance-related outcomes, empirical evidence has shown that self-leadership affects employees’ work performance positively (Griffin et al., 2007), enhancing their task proficiency, adaptability, and proactivity (Hauschildt and Konradt, 2012; Marques-Quinteiro and Curral, 2012; Bailey et al., 2018; Marques-Quinteiro et al., 2019). On the other hand, the literature is limited in terms of the theory-driven, empirical studies (e.g., Panagopoulos and Ogilvie, 2015; Inam et al., 2021) that explore the mechanisms through which self-leadership nurtures different aspects of work role performance. Indeed, although previous research has focused on a diverse group of variables (i.e., self-efficacy, work engagement, job satisfaction) that link self-leadership to performance, the motivational, self-empowerment process starting with self-leadership and leading to increased work role performance is under-researched and warrants empirical examination (Goldsby et al., 2021), particularly in remote work settings.

Moreover, despite the burgeoning research interest in the impact of self-leadership on different performance outcomes, and with a few notable exceptions, there is a dearth of knowledge about the boundary conditions that may affect the self-leadership—work role performance relationship. Panagopoulos and Ogilvie (2015) considered organization-based self-esteem as a boundary condition that positively moderated the indirect relationship between self-leadership and salesperson performance. In a more recent study, Kalra et al. (2021) positioned technical knowledge as a moderating factor that alleviated the linkages between behavioral self-leadership and adaptive selling behaviors as well as sales performance. While these studies drew attention to the importance of boundary conditions in self-leadership research, they focused primarily on personal factors (e.g., employee characteristics or competencies) in shaping the linkages between self-leadership and performance outcomes. In doing so, they overlooked the moderating role of external factors, particularly those associated with supervisor control, on the employee initiated self-empowerment process. Indeed, some recent studies have investigated the direct effects of external factors, such as leader’s motivating language, on different self-leadership behaviors (Mayfield et al., 2021; Mayfield and Mayfield, 2021). However, less is known about whether and how external contingencies such as supervisor control might shape the motivational process started with self-leadership and leading to enhanced work role performance.

A major gap in the self-leadership research arises from the work environments in which prior studies have been conducted, primarily traditional physical working conditions which involve face-to-face communication and human interaction (e.g., Konradt et al., 2009; Hauschildt and Konradt, 2012; Marques-Quinteiro and Curral, 2012). The growth of physically distant working conditions (e.g., telework or hybrid work), which has resulted in greater flexibility and autonomy in daily working routines (Müller and Niessen, 2019), necessitates further investigation of the effects of self-leadership in such work contexts. Surprisingly, few attempts (Castellano et al., 2021; Costantini and Weintraub, 2022) have been made to evaluate the outcomes of self-leadership in remote or hybrid working contexts, which might in fact serve as ideal settings to study the effects of “leading oneself” toward task accomplishment (Manz, 1986). Although previous research has demonstrated a link between self-leadership and employee performance in remote work settings (Castellano et al., 2021; Costantini and Weintraub, 2022), these studies focused on a single aspect of work role performance, i.e., task performance or proactivity, among remote workers. Further, only a limited research effort has been directed toward understanding the boundary conditions that may affect the performance outcomes of self-leaders who are working remotely. A more comprehensive approach is thus needed to empirically scrutinize the impacts of self-leadership on different performance outcomes in specific contexts such as remote working, and under different external contingencies.

**Figure 1:**
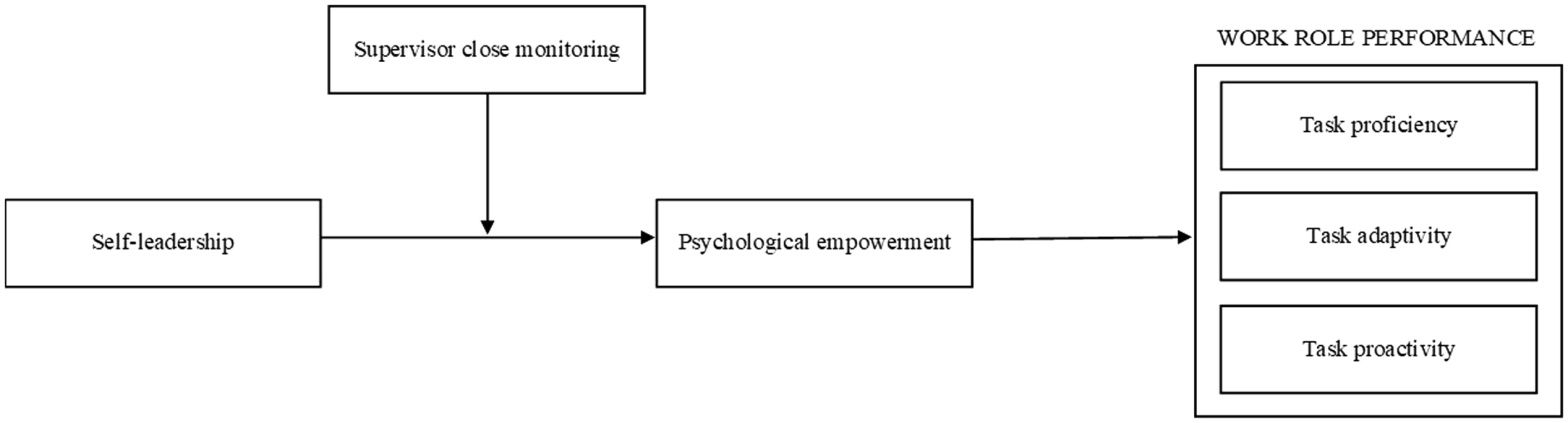
Conceptual model.

Against this background, we seek to make the following contributions to the self-leadership and work role performance literatures. First, drawing on self-determination theory (SDT; Deci et al., 2017) and Zimmerman (1995, 2000) empowerment theory, we develop and test an integrative model that demonstrates the motivational process through which self-leadership leads to increased work role performance (Figure 1). Specifically, we suggest that psychological empowerment might act as a dynamic, autonomous motivational state that links self-leadership to positive performance outcomes, including increased task proficiency, adaptivity, and proactivity. Second, we position supervisor close monitoring as an external control factor which shapes the motivational and performance outcomes of self-leadership. Finally, we test our integrative model in a relatively under-researched work setting, remote work, which is a practice that has taken widespread root among many organizations during and after the COVID-19 pandemic. Exploration of the proposed set of relationships under remote work conditions is noteworthy as it advances our understanding of the possible interactive effects of self-regulation/control (i.e., self-leadership) and external regulation/control (i.e., supervisor close monitoring) in a unique setting that may genuinely pave the way for both (virtual) self-leadership and supervisors’ close monitoring. It also provides valuable practical insights into how employees with self-leadership capabilities need to be approached and managed for better performance outcomes under remote work settings.

## Background and hypotheses development

### Self-leadership and work role performance

Self-leadership refers to a set of cognitive and behavioral actions through which individuals attain self-motivation and self-direction that enhance their overall performance (e.g., Manz, 1986; Neck and Houghton, 2006). These strategies can be grouped under three major categories: (1) *Behavior-focused strategies* (e.g., self-observation and self-goal setting), (2) *Constructive thought pattern strategies* (e.g., evaluating beliefs and assumptions and visualizing successful performance), (3) *Natural reward strategies* (e.g., focusing on natural rewards; Houghton and Neck, 2002).

According to previous research, one of the primary outcomes associated with self-leadership is enhanced work role performance (e.g., Hauschildt and Konradt, 2012; Marques-Quinteiro and Curral, 2012; Bailey et al., 2018). Work role performance is a multifaceted concept that involves individual-, team-, and organization-level work role behaviors. For the scope of our research, we focus only on individual task role behaviors, namely task proficiency, task adaptivity, and task proactivity. *Task proficiency* describes the extent to which an employee fulfills the predefined requirements of his or her work role (Griffin et al., 2007). *Task adaptivity* characterizes the degree to which individuals contend with and adapt to changes that influence their roles in dynamic work settings (Griffin et al., 2007). Finally, *task proactivity* describes the extent to which individuals perform self-initiated, change-oriented behaviors to shape their work roles and change themselves in uncertain work environments (Griffin et al., 2007).

Empirical research has shown that self-leadership is positively associated with different aspects of work-role performance (Marques-Quinteiro and Curral, 2012; Bailey et al., 2018; Marques-Quinteiro et al., 2019). On the other hand, the literature is limited in terms of theory-driven, empirical studies that examine the interlinking mechanisms between self-leadership and work-role performance. The extant research focused on self-efficacy (Chaijukul, 2010; Panagopoulos and Ogilvie, 2015), job satisfaction (Chaijukul, 2010), and work engagement (Inam et al., 2021) as three mediating mechanisms that link self-leadership into work-role performance. Relying on SDT (Deci et al., 2017) and Zimmerman (1995, 2000) empowerment theory, we suggest that psychological empowerment might also serve as an overarching motivational mechanism through which self-leadership behaviors are translated into better work role performance. We explain this process in greater detail below.

### Mediating role of psychological empowerment

Psychological empowerment refers to “a motivational construct manifested in four cognitions: meaning, competence, self-determination, and impact” (Spreitzer, 1995, 1,443). Based on these four cognitions, psychologically empowered individuals feel a kind of autonomous motivation at work as they seek and give meaning to their job, feel capable of performing the job, find ways to be autonomous, and recognize the impact of their job on the overall performance of the department or organization. In his seminal work on empowerment theory, Zimmerman (1995, 2000) has suggested that psychological empowerment involves “beliefs that goals can be achieved, awareness about resources and factors that hinder or enhance one’s efforts to achieve those goals, and efforts to fulfill the goals” (p. 582).

Studies exploring the link between self-leadership and empowerment assert that self-leadership strategies such as self-goal setting, self-reward, and visualizing successful performance enhance employees’ psychological empowerment at work by fostering feelings of self-determination, competence, meaning/purpose, and impact (Manz, 1992; Houghton and Yoho, 2005; Chaijukul, 2010; Amundsen and Martinsen, 2015). This argument has also found support in psychological empowerment theory (Zimmerman, 1995, 2000), which implies that self-leadership skills empower employees by helping them become autonomous, enabling them to control the events in their lives (including their work lives), and guiding them to become their own best supporters toward the achievement of goals. As such, these skills or strategies are likely to enhance employees’ autonomous motivation which is one of the two major types of motivation according to Deci et al (2017) basic self-determination theory (SDT) model for the workplace.

According to Deci et al. (2017), there are two major types of motivations that predict various workplace outcomes including employee performance. The first is labeled as *autonomous motivation* that describes “the process of being motivated by one’s interest in an activity (i.e., intrinsic motivation) and/or the value and regulation of the activity internalized within the self (i.e., integrated extrinsic motivation)” (Sun et al., 2012, 57). In contrast, *controlled motivation*, as the second motivation type, refers to a form of external regulation, in which individuals’ behavior is shaped by the external conditions in the work setting such as rewards, punishments, or power dynamics (Deci et al., 2017). SDT also postulates three primary needs (autonomy, competence, and relatedness), the satisfaction of which promotes the experience of autonomous motivation. These needs are conceptually in line with the four cognitions underlying psychological empowerment (i.e., meaning, self-determination, competence, and impact; Morin et al., 2016), representing an ideal form of autonomous motivation in the work setting.

In this study, we suggest that psychological empowerment is likely to act as a dynamic, motivational mechanism that links self-leadership to the three distinct aspects of work role performance (Cerasoli et al., 2014). Specifically, we propose that the self-leadership strategies or behaviors are likely to initiate an employee-driven, self-empowerment process through which employees develop a sense of autonomous motivation in their job that eventually enhances their work role performance. That is, employees pursuing self-leadership strategies develop a sense of perceived control, competence, and mastery of their work domain (i.e., psychological empowerment; Zimmerman, 1995), which might improve their task proficiency, adaptability, and proactivity. Prior studies have revealed that psychological empowerment enhances employees’ task proficiency by increasing their concentration, persistence, resilience, and effort at work (Thomas and Velthouse, 1990; Bonias et al., 2010). It also spurs employees into a more active rather than passive role as they execute their job responsibilities, leading to superior adaptive and proactive performance (Zhang et al., 2017; Xu and Zhang, 2022). Accordingly, psychological empowerment does not only help employees perform their job requirements adequately (i.e., task proficiency) but also releases their potential to adapt to the changes (i.e., task adaptivity) and to take the initiative and make positive changes in their work roles (i.e., task proactivity; Martin et al., 2013). Based on the above, we propose that:

*Hypothesis* 1: Psychological empowerment mediates the positive relationship between self-leadership and task (a) proficiency, (b) adaptivity, and (c) proactivity.

### Moderating role of supervisor close monitoring

Supervisor close monitoring refers to a type of external control through which supervisors “keep close tabs on their subordinates to ensure that they do what they are told, perform tasks in expected ways, and do not do things that the supervisor might disapprove of” (George and Zhou, 2001, 515). This type of supervisory behavior represents a type of external regulation which signals to employees that they need to act in line with the rules and expectations set by the organization. Under supervisor close monitoring, employees are likely to feel that they are being regularly monitored and controlled by their supervisor (George and Zhou, 2001; Zhou, 2003).

Prior research assigns a predominantly negative connotation to supervisor close monitoring (Son et al., 2017; Lebel and Patil, 2018; Kim, 2019) although there are a handful of studies suggesting that close monitoring might also create positive outcomes when it helps employees satisfy their primary psychological needs (e.g., relatedness and competence; Mishra and Ghosh, 2020). The level and impact of close monitoring is deemed particularly controversial in remote working conditions. On the one hand, direct observation or monitoring by supervisors is likely to be limited under remote work, which provides little room for real-time control of employees’ task-focused behaviors (Gong and Sims, 2023). On the other hand, when managers are unable to monitor employee performance directly, they may feel distressed that employees disregard task-oriented behaviors (Whitener et al., 1998; Kurland and Cooper, 2002; Dimitrova, 2003) and thus they may increase their surveillance and control in the remote work setting (Sitkin and Roth, 1993; Forbes, 2021; Gan et al., 2022). As such, it is possible to observe both low and high supervisory control (in the form of close monitoring) under remote working conditions.

In this study, we propose that supervisor close monitoring will affect the motivational, self-empowering process starting with self-leadership and leading to better work role performance negatively. As discussed previously, employees following self-leadership strategies tend to feel more autonomously motivated (i.e., psychologically empowered) in their job. On the contrary, when these employees are closely monitored, their psychological empowerment might decline as the external control and regulation by the supervisor deteriorates their sense of self-determination, strongly clashing with the self-management nature of self-leadership.

Specifically, under supervisor close monitoring, employees tend to work primarily to avoid punishment and criticism (Son et al., 2017) and thus show less effort to go beyond the job requirements (Rietzschel et al., 2014; Lee et al., 2019). Feeling a lack of autonomy and impact in their job, self-leaders will be particularly reluctant or demotivated to perform their job duties proficiently. Similarly, close monitoring might weaken these employees’ sense of control and competency in their job (Kim, 2019) and impair their self-regulation resources (Lee et al., 2019), limiting their adaptivity. When self-leaders know that their performance is closely tracked, they will find it difficult and even risky (Lebel and Patil, 2018) to immediately adapt to new situations—unless they believe that their supervisor will approve the way they act. Finally, supervisor close monitoring is likely to discourage self-leaders’ voluntary and autonomous motivation to engage in proactive actions (Son et al., 2017) as these might contradict the predetermined rules and regulations and thus prompt their supervisor’s disapproval. Although self-leadership strategies tend to stimulate feelings of autonomy or discretion, which have been discussed as the preconditions for proactivity at work (e.g., Grant and Ashford, 2008; Bindl and Parker, 2010), excessive external control and regulation by supervisors might alleviate this triggering effect. In line with the previous arguments, we hypothesize:

*Hypothesis* 2: Supervisor close monitoring moderates the indirect effect of self-leadership on individual task (a) proficiency, (b) adaptivity, and (c) proactivity through empowerment such that this effect is weaker (stronger) when supervisor close monitoring is high (low).

We tested the previous hypotheses in two different studies. In Study 1, we shed light on the relationships between self-leadership, psychological empowerment, and work role performance by implementing a time-lagged design. In Study 2, we tested our theoretical model (Figure 1), which also involved the moderating effect of supervisor close monitoring, with a cross-lagged two wave design.

## Methodology (study 1)

### Sample and procedure

We collected data from a sample of 174 white-collar employees from a broad range of jobs and organizations who work in Istanbul, Turkey. Participants were obtained *via* standardized recruitment messages in professional and social networking sites (LinkedIn and Twitter), personal networks of the researchers, and snowball sampling. As we aimed to test our model primarily in the remote working context, we required that participants work at least 23 h or 3 days a week from home to be included in the study, considering that under Turkish Labor Law, regular full-time workers work at least 45 h a week. Participants were also expected to have minimum face-to-face interaction with their supervisors and coworkers when they were working in the office. As such, only those individuals (a) who worked at least 23 h or 3 days a week from home and (b) who either did not interact or interacted minimally with their supervisors (and coworkers) in the office received an online survey through which they evaluated their self-leadership strategies and psychological empowerment (Time 1). One week after the initial survey, employees completed a second survey, which included questions about their task proficiency, adaptivity, and proactivity (Time 2). Data collection commenced in April 2020, when majority of the white-collar employees in Turkey were working remotely due to the health and safety precautions for COVID-19 pandemics,^1^ and lasted for 1 month.

Initially, 226 employees were contacted, 210 of whom agreed to participate in our study. After eliminating the incomplete survey forms (i.e., forms with unanswered questions) and those dropped out of the sample in the second week of data collection, we had the full data for 174 employees, representing a response rate of 77%. 98% of the employees were working remotely (at home or at another location away from the employer’s location) at least 23 h or 3 days a week. The remaining 2% were fieldworkers (who can be also considered remote workers). Participants’ average age was 39.95 years (SD = 6.99), and their average job tenure and work experience were 8.15 years (SD = 6.12) and 14.39 years (SD = 7.67), respectively. Among these employees, 62% were females and majority of the participants (97%) had an undergraduate or graduate degree. Finally, employees in the sample were working in various sectors (e.g., banking and finance, education, consultancy) and departments (e.g., strategy/business development, human resources management), increasing the generalizability of our study.

### Measures

We first developed the survey forms in Turkish and then translated the questions into Turkish in line with Brislin (1986) back-translation method. All the items were assessed with 5-point Likert scales, as explained in more detail below.

#### Self-leadership

We used the Abbreviated Self-Leadership Questionnaire (ASLQ; Houghton et al., 2012) to assess employees’ self-leadership practices. The ASLQ is a nine-item, condensed version of the 35-item Revised Self-Leadership Questionnaire (RSLQ; Houghton and Neck, 2002), which has good reliability and validity in comparison to the original RSLQ (Nel and van Zyl, 2015; Mahembe et al., 2017). Sample items are: “I work toward specific goals I have set for myself,” and “Sometimes I picture in my mind a successful performance before I actually do a task.” The rating scheme involved 1 = strongly disagree to 5 = strongly agree (α = 0.81).

#### Psychological empowerment

We measured psychological empowerment with Spreitzer (1995) 12-item empowerment scale, based on four cognitions: meaning, competence, self-determination, and impact. Sample items involve: “The work I do is very important to me” (meaning; α = 0.91), “I am confident about my ability to do my job”; (competence; α = 0.91), “I can decide on my own how to go about doing my work” (self-determination; α = 0.88), and “I have significant influence over what happens in my department” (impact; α = 0.94). The rating scale ranged from 1 = strongly disagree to 5 = strongly agree (α = 0.92).

#### Work role performance

We adopted Griffin et al. (2007) work role performance scales to evaluate employees’ work role performance. Participants were requested to evaluate the extent to which they had performed the respective behaviors over the last month, e.g., “Carried out the core parts of his/her job well” (task proficiency), “Adapted well to changes in core tasks” (task adaptivity), and “Come up with ideas to improve the way in which his/her core tasks are done” (task proactivity). They provided their answers on a scale ranging from “very little/none” (1) to “a great deal” (5). Reliabilities were satisfactory (0.84, 0.81, and 0.87, respectively) for all three aspects of work role performance.

#### Controls

Both theoretical and empirical evidence revealed that there is an effect of gender, experience, and tenure on employees’ task proficiency (e.g., Griffin et al., 2007; Avey et al., 2010), task adaptivity (e.g., García-Chas et al., 2015; Wu et al., 2017), and task proactivity (e.g., Thomas et al., 2010). Hence, we incorporated gender (0 = female, 1 = male), work experience, and tenure as control variables that may influence our outcome variables.

### Preliminary analyses

We performed several confirmatory factor analyses (CFAs) with AMOS 27 to examine the fit scores of the overall measurement model (in which all five constructs were separately represented) and test the distinctiveness of the constructs. Specifically, we compared the relative fit of four-, three-, two-, and single-factor models to the five-factor measurement model. We also checked the relative fit of the common-method factor model against a model involving self-reported items (i.e., self-leadership and psychological empowerment; see Appendix Table A1 for details).^2^ In all CFAs, the five-factor model demonstrated better fit than the alternative models [χ^2^(197) = 340.86, *p* < 0.01, CFI = 0.92, TLI = 0.91, RMSEA = 0.07, RMR = 0.05, SRMR = 0.07]. Harman’s single-factor test also demonstrated a very poor fit [χ^2^(207) = 552.32, *p* < 0.01, CFI = 0.63, TLI = 0.59, RMSEA = 0.14, and SRMR = 0.12]. Common-method factor model was created such that the measured items did not only load on their conceptual factors, respectively, but also loaded on a single method factor. In this way, unobservable sources of common method variance can be integrated to the model as latent factors (Williams and Anderson, 1991). The findings showed that the model that involved a single (common) method factor revealed lower fit scores than the two-factor model (i.e., the model with two independent factors, i.e., self-leadership and psychological empowerment).

We further tested the convergent and discriminant validity of the self-reported measures (i.e., self-leadership and psychological empowerment) by checking the factor loadings, average variance extracted (AVE), and the shared variance between constructs. All factor loadings were significant ranging from 0.50 to 0.88 and 0.66 to 0.76 for self-leadership and psychological empowerment, respectively. Moreover, AVE values exceed 0.50 for both constructs (i.e., 0.53 for self-leadership and 0.51 for psychological empowerment) further validating their convergent validity (Fornell and Larcker, 1981). The discriminant validity was also verified as the AVE of each construct was greater than the square of the correlation (ρ^2^ = 0.24) between constructs (Fornell and Larcker, 1981). Heterotrait-monotrait (HTMT) criterion test validated this finding by showing that the correlation between constructs (r = 0.49) were less than the cut-off value of 0.90 (Henseler et al., 2015).

Overall, the findings showed that the respondents could distinguish the five self-reported measures well, and common method variance was not a critical concern for the subsequent analyses.

## Results (study 1)

Table 1 demonstrates the means, standard deviations, reliabilities, and correlations for the main variables.

**Table 1:** Descriptives and correlations between variables (Study 1).

		Mean	SD	1	2	3	4	5	6	7	8
1	Gender	0.40	0.49	-							
2	Tenure	8.15	6.12	0.10	-						
3	Work experience	14.39	7.67	0.01	0.62^**^	-					
4	Self-leadership	3.78	0.61	−0.06	−0.15^*^	(0.81)					
5	Psychological empowerment	3.63	0.76	0.08	0.18^*^	0.26^**^	0.35^**^	(0.92)			
6	Task proficiency	3.92	0.73	−0.05	0.06	0.10	0.21^**^	0.36^**^	(0.84)		
7	Task adaptivity	3.81	0.72	−0.03	0.04	0.07	0.33^**^	0.34^**^	0.68^**^	(0.81)	
8	Task proactivity	4.19	0.67	0.02	−0.05	−0.04	0.37^**^	0.39^**^	0.38^**^	0.64^**^	(0.87)

*n* = 174. Gender: “0” = Female, “1 = Male. Reliability coefficients are in parentheses.

* *p* < 0.05;

** *p* < 0.01.

We tested the relationships in our mediation model by applying structural equation modeling (SEM) methodology with AMOS 27. The results revealed that the model had a reasonable fit to the data [χ^2^(255) = 446.27, *p* < 0.05; CFI = 0.90; TLI = 0.88; RMSEA = 0.07; and SRMR = 0.08; Mulaik et al., 1989; Hu and Bentler, 1999]. Moreover, the results showed that after controlling performance outcomes for gender, tenure, and experience, self-leadership was positively related to psychological empowerment (*β* = 0.53, *p* < 0.01), and the effect of empowerment on task proficiency (*β* = 0.51, *p* < 0.01), task adaptivity (*β* = 0.28, *p* < 0.05), and task proactivity was also significant (*β* = 0.41, *p* < 0.05). Given these results, we also checked the indirect effects of self-leadership on three different aspects of work role performance. We calculated the confidence intervals (CIs) of the indirect effects by performing bootstrapping (specifying 5,000 replications) in the AMOS 23 program. As shown in Table 2, the results revealed a significantly positive indirect impact of self-leadership on (a) task proficiency [*β* = 0.27, p < 0.01, 95% CI = (0.09, 0.68)], (b) task adaptivity [*β* = 0.15, *p* < 0.10, 95% CI = (0.00, 0.54)], and (c) task proactivity [*β* = 0.22, p < 0.01, 95% CI = (0.06, 0.54)]. These findings provided initial support for Hypothesis 1.

**Table 2:** Mediation results (Study 1).

Outcome: Task proficiency				Outcome: Task adaptivity				Outcome: Task proactivity			
**Direct and indirect effects and 95% confidence intervals**											
**Standardized direct effects**				**Standardized direct effects**				**Standardized direct effects**			
**Parameter**	**Estimate**	**Lower**	**Upper**	**Parameter**	**Estimate**	**Lower**	**Upper**	**Parameter**	**Estimate**	**Lower**	**Upper**
SL → PE	0.53^**^	0.22	0.77	SL → PE	0.53^**^	0.22	0.77	SL → PE	0.53^**^	0.22	0.77
SL → TProf	0.04	−0.31	0.37	SL → TAdapt	0.28	−0.07	0.59	SL → TPro	0.23	−0.08	0.49
PE → TProf	0.51^**^	0.25	0.87	PE → TAdapt	0.28^*^	0.02	0.76	PE → TPro	0.41^**^	0.15	0.72
**Standardized indirect effects**				**Standardized indirect effects**				**Standardized indirect effects**			
SL → PE → TProf	0.27^*^	0.09	0.68	SL → PE → TAdapt	0.15^*^	0.00	0.54	SL → PE → TPro	0.22^**^	0.06	0.54

*n* = 135 (bootstrapping by specifying a sample of size 5,000). SL, self-leadership; PE, psychological empowerment; TProf, task proficiency; TAdapt, task adaptivity; TPro, task proactivity.

* *p* < 0.05;

** *p* < 0.01.

## Methodology (study 2)

### Sample and procedure

In Study 2, we gathered data from 135 employees and their supervisors working in small and medium-sized firms in Istanbul, Turkey. An independent research firm, which had a well-established SME network in different sectors, conducted the data collection process as part of a wider research project examining the correlates of self-leadership. As in Study 1, employees who worked at least 23 h or 3 days a week from home and had minimum interaction with their supervisors and coworkers in the office environment were included in the study. Data collection started in June 2020 and lasted for 2 months.

The survey data were collected through an online survey system at two time points (2 weeks apart). At Time 1, SME employees were asked to answer questions about their self-leadership behaviors, psychological empowerment, and demographics. They also evaluated their supervisors’ monitoring behaviors. Two weeks later (Time 2), they were asked to answer the same questions, using the measures employed at Time 1. Moreover, at Time 2, supervisors were requested to evaluate the work role performance along three behavioral dimensions: task proficiency, task adaptivity, and task proactivity. At Time 1, surveys were distributed to 200 SME employees *via* the online survey system. 160 employees filled the online survey forms at Time 1, with a response rate of 80%. Of these, 150 employees completed the Time 2 surveys, indicating a response rate of 94%. At the same period, we received 140 matching supervisor surveys. Of these, five cases were dropped as they included missing ratings for at least two or more of the performance dimensions. Excluding these, 135 complete surveys were used in the analyses.

Among the employees, the average age was 36.17 years (SD = 8.82), and the mean job tenure and experience were 3.04 years (SD = 2.57) and 9.65 years (SD = 6.76), respectively. Females constituted 51% of the overall sample. Respondents primarily had a university degree (54%), followed by high school (33%), and graduate degrees (13%). As in Study 1, employees in the sample were working in various sectors (e.g., food and beverage, computer/technology, real-estate) and departments (e.g., finance and accounting, operations, and marketing), holding different formal positions (i.e., managers/non-managers). In our sample, employees from the same organization were reporting to a single (the same) supervisor.

### Measures

Following the same procedure with Study 1, all the scales went through a translation and back-translation process and were measured by 5-point Likert scales. For the self-leadership, psychological empowerment, and work role performance (i.e., task proficiency, task adaptivity, and task proactivity), we used the same measures previously described in Study 1. The internal consistency reliabilities of these measures in the Study 2 ranged from 0.72 to 0.90. In addition, for Study 2, we incorporated supervisor close monitoring using the following measure.

#### Supervisor close monitoring

Supervisor close monitoring was measured by the 6-item scale developed by George and Zhou (2001). Participants (employees) were asked to evaluate their supervisor’s close monitoring behavior using George and Zhou (2001) six-item measure (see Appendix). The rating scheme involved 1 = strongly disagree to 5 = strongly (α = 0.83).

As in Study 1, we controlled for the effects of employees’ gender, experience, and tenure to avoid possible confounding effects.

### Preliminary analyses

Given that self-leadership, psychological empowerment, and supervisor close monitoring were rated by the employees themselves, we conducted several CFAs with AMOS 27 to examine whether employee scores on self-reported measures denoted idiosyncratic constructs. For all CFAs, we included psychological empowerment as a higher-order factor as the higher-order model fitted the data better than the single-factor model [∆χ^2^(4) = 53.98, *p* < 0.01] and the orthogonal first-order model [∆χ^2^(4) = 242.45, *p* < 0.01]. The higher-order model showed a comparable fit to the oblique first-order model [∆χ^2^(2) = 1.12, *p* < 0.01].

The findings revealed an acceptable fit for the hypothesized three-factors (Time 1) and two-factors (Time 2) structure, for the data collected at two-time phases. For the Time 1 data, the hypothesized structure, where self-leadership, psychological empowerment, and supervisor close monitoring constituted the three different factors, had a reasonable fit [χ^2^(101) = 168.47, CFI = 0.94; RMSEA = 0.07; SRMR = 0.05; Mulaik et al., 1989; Hu and Bentler, 1999]. On the other hand, the alternative models, including the single factor model had a poorer fit [Δχ^2^(3) = 216.49, *p* < 0.01]. The findings also showed that the model that involved a single (common) method factor revealed lower fit scores than the three-factors model (i.e., the model with three independent factors for self-leadership, psychological empowerment, and supervisor close monitoring) We observed the same pattern for the self-reported Time 2 data (i.e., self-leadership and psychological empowerment). Specifically, the model where self-leadership and psychological empowerment were represented by two different factors, had a good fit [χ^2^(34) = 68.10, CFI = 0.94; RMSEA = 0.09; SRMR = 0.05; Hu and Bentler, 1999; Mulaik et al., 1989] while the alternative models (i.e., single factor model and the two-factors model with a common-factor) had a poorer or at least a similar fit.^3^

We further tested the discriminant validity among the self-reported constructs by checking whether the average variance extracted (AVE) for each construct was (at Time 1 and Time 2) greater than its shared variance with any of the other constructs (Fornell and Larcker, 1981). The discriminant validity was verified for Time 1 data as the AVE of each construct (i.e., 49%, 61%, and 44% for self-leadership, psychological empowerment, and supervisor close monitoring, respectively) was greater than the square of the correlation (ρ^2^) between that specific construct and any others. Although the AVE values for self-leadership and psychological empowerment at Time 2 were slightly lower than the square of the correlation between these constructs (Fornell and Larcker, 1981), Heterotrait-monotrait (HTMT) criterion test conveyed discriminant validity by showing that the correlation between constructs (r = 0.84) were less than the cut-off value of 0.90 (Henseler et al., 2015).

Additionally, we performed multi-group CFAs to confirm the measurement equivalence of both self-leadership and psychological empowerment across the two-time frames (Vandenberg and Lance, 2000). First, we ran a multi-group CFA in which the self-leadership item loadings were set as identical across Times 1 and 2. This model fitted the data reasonably well, χ^2^(23) = 102.59, *p* < 0.01; CFI = 0.88; SRMR = 0.07, denoting a configural invariance of self-leadership measures across the two time points. The multi-group CFA also confirmed the configural invariance of psychological empowerment measures across two time points [χ^2^(7) = 17.66, *p* < 0.01; CFI = 0.98; SRMR = 0.03]. Taken together, the findings showed that the factorial structure and item loadings of self-leadership and psychological empowerment remained the same across two periods. Thus, we could investigate the relationships among these constructs measured at two distinct time points.

### Analytic strategy for the mediation hypothesis

**Figure 2:**
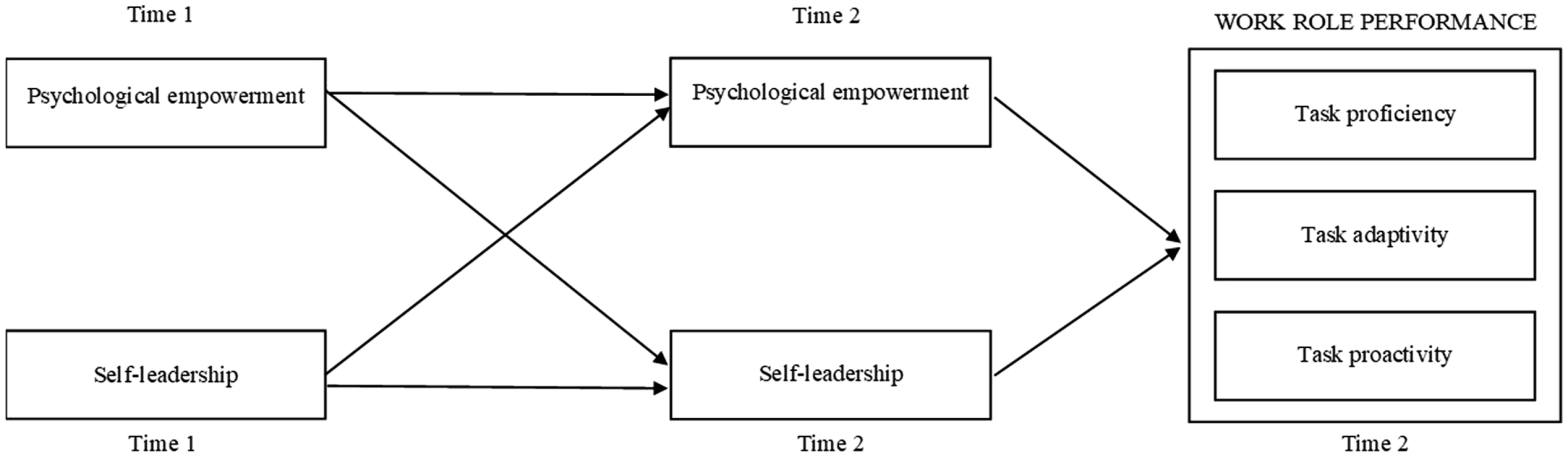
Cross-lagged model.

We collected the data of SME employees’ self-leadership and psychological empowerment in both Time 1 and Time 2 and employed a cross-lagged panel data design with AMOS 27 (Selig and Preacher, 2009) to examine the nature of the relationship between self-leadership and psychological empowerment. Figure 2 depicts our cross-lagged model in which work role performance dimensions (measured in Time 2) were positioned as the outcome of self-leadership and psychological empowerment. To test our hypotheses, we followed the procedure described by Zhang et al. (2016).

Because both self-leadership and psychological empowerment are based on employee perceptions and there are few empirical studies which suggested empowerment as an antecedent of self-leadership (e.g., Wilson, 2011), it may be problematic to make clear inferences whether self-leadership triggers psychological empowerment, or the reverse is also true. The cross-lagged design may help solve this problem by testing both directions of causality at the same time while controlling for the impact of each variable at a previous time (Zhang et al., 2016). Specifically, while simultaneously testing the relationship between Time 1 self-leadership and Time 2 psychological empowerment and Time 1 empowerment and Time 2 self-leadership, the effects of Time 1 self-leadership and Time 1 psychological empowerment on their Time 2 equivalents were also considered.

## Results (study 2)

Table 2 demonstrated the descriptive statistics and inter-correlations for the study variables.

### Mediation of psychological empowerment

**Figure 3:**
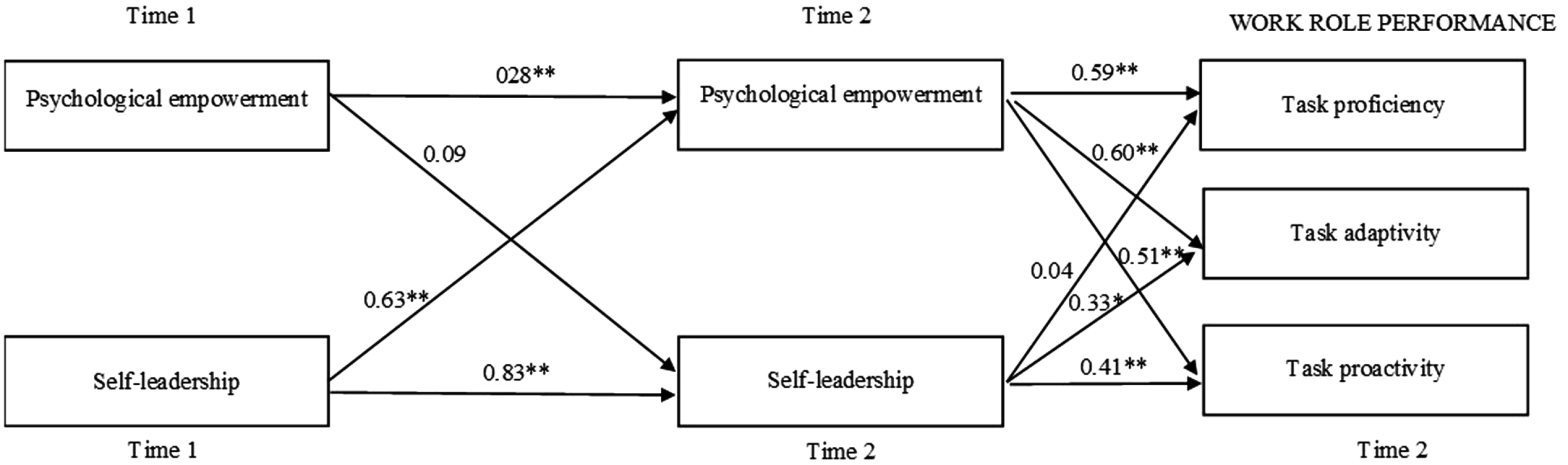
Cross-lagged model with path coefficients. ^**^p < 0.01, ^*^p < 0.05.

Results revealed that after controlling for possible reverse causation (i.e., psychological empowerment at Time 1 affects self-leadership at Time 2), self-leadership affected psychological empowerment significantly (*β* = 0.63, *p* < 0.01), and the effects of empowerment on task proficiency (*β* = 0.59, *p* < 0.01), task adaptivity (*β* = 0.60, *p* < 0.01), and task proactivity (*β* = 0.51, *p* < 0.01) were also significant (Figure 3).

Given these results, we estimated the indirect effects of self-leadership on work role performance through empowerment by testing the product of two parameters, path estimate from self-leadership to empowerment, and path estimate from empowerment to the respective performance outcome. We checked the confidence interval of these indirect effects using the bootstrapping procedure. Our results showed that, with 5,000 bootstrapping replications, there was a significant indirect effect of self-leadership on (a) task proficiency [*β* = 0.26, *p* < 0.01, 95% CI = (0.05, 0.82)], (b) task adaptivity [*β* = 0.27, *p* < 0.01, 95% CI = (0.08, 0.75)], and (c) task proactivity [*β* = 0.26, *p* < 0.01, 95% CI = (0.07, 0.77)]. Taken together, these findings provided support for our Hypothesis 1.

### Moderation of the indirect effect

To test the moderating effect of supervisor close monitoring on the mediation model, we performed multi-group analysis and χ^2^ difference test (specifying a bootstrapping sample of 5,000 at a 95% confidence interval). For this purpose, we divided the sample into two groups (one high and one low on the moderator, that is, supervisor close monitoring) with a median split procedure. We conducted a separate multi-group analysis for each performance outcome. In each analysis, we checked a Chi-square difference between the constrained model in which hypothesized affected were constrained to be equal across the low and high groups and an unconstrained model in which the same paths varied freely across two groups. The moderating hypothesis was verified if the unconstrained model demonstrated a significantly lower chi-square than the constrained model.

The results showed that for the performance outcomes of task proficiency, task adaptivity, and task proactivity, χ^2^ difference between the unconstrained model (i.e., the model in which none of the structural paths was constrained for the equality of their weights) and constrained model (i.e., the path between self-leadership and psychological empowerment was set to be equal across two groups) was significant (model with task proficiency: Δχ^2^(1) = 18.83, *p* < 0.01; model with task adaptivity: Δχ^2^(1) = 18.05, *p* < 0.01, and model with task proactivity: Δχ^2^(1) = 17.77, *p* < 0.01). This suggests that supervisor close monitoring is likely to moderate the indirect effect of self-leadership on the work role performance.

The bootstrapping results of moderated models for both low close monitoring and high close monitoring groups are presented in Table 3. As shown in this table, multi-group models displayed a good fit with the data. These results showed that the indirect effect of self-leadership on task proficiency was non-significant for employees who experienced low supervisor close monitoring in their job as well as for those who were closely monitored by their supervisors. Thus, Hypothesis 2a was not supported.^4^ Further, in support of Hypothesis 2b, the results revealed that the indirect effect of self-leadership on task adaptivity was significant only for those employees who were loosely monitored by their supervisors [*β* = 0.54, *p* < 0.05, 95% CI = (0.10, 3.08)]. Similarly, the indirect relationship between self-leadership and task proactivity was significant only for those employees who were not closely monitored by their supervisors [*β* = 0.51, *p* < 0.5, 95% CI = (0.4, 4.01)]. Hence, Hypothesis 2b and 2c were supported (Table 4).

**Table 3:** Decriptives and correlations between variables (Study 2).

		*Mean*	*SD*	1	2	3	4	5	6	7	8	9	10	11
1	Gender	0.50	0.50	-										
2	Tenure	3.28	3.21	−0.01	-									
3	Work experience	9.67	6.82	0.10	0.47^**^	-								
4	T1 Self-leadership	3.56	0.64	−0.01	0.04	−0.18^*^	(0.85)							
5	T1 Psychological empowerment	3.37	0.83	0.09	0.01	−0.06	0.58^**^	(0.92)						
6	T2 Self-leadership	3.52	0. 71	−0.06	0.02	−0.17^*^	0.93^**^	0.56^**^	(0.86)					
7	T2 Psychological empowerment	3.67	0.59	0.03	−0.11	−0.14	0.65^**^	0.67^**^	0.59^**^	(0.90)				
8	Supervisor close monitoring	3.32	0.81	−0.04	0.10	−0.04	0.40^**^	0.53^**^	0.38^**^	0.48^**^	(0.83)			
9	Task proficiency	3.41	0.89	−0.15	0.01	−0.09	0.40^**^	0.38^**^	0.33^**^	0.51^**^	0.34^**^	(0.74)		
10	Task adaptivity	3.48	0.82	−0.03	0.19^*^	0.03	0.57^**^	0.47^**^	0.48^**^	0.52^**^	0.44^**^	0.64^**^	(0.75)	
11	Task proactivity	3.27	0.79	−0.17	−0.01	−0.13	0.40^**^	0.36^**^	0.37^**^	0.45^**^	0.34^**^	0.79^**^	0.52	(0.72)

*n* = 135. Gender: “0” = Female, “1 = Male. Reliability coefficients are in parentheses. T1, Time 1; T2, Time 2.

* *p* < 0.05;

** *p* < 0.01.

**Table 4:** Moderated mediation results.

Outcome: Task proficiency (H1a) model fit: χ^2^(192) = 269.19, CFI = 0.90; RMSEA = 0.06; SRMR = 0.07				Outcome: Task adaptivity (H1b) model fit: χ^2^(192) = 256.40, CFI = 0.91; RMSEA = 0.05; SRMR = 0.07				Outcome: Task proactivity (H1c) model fit: χ^2^(192) = 289.53, CFI = 0.88; RMSEA = 0.06; SRMR = 0.07			
**Direct and indirect effects and 95% confidence intervals—Low close monitoring**				**Direct and indirect effects and 95% confidence intervals—Low close monitoring**				**Direct and indirect effects and 95% confidence intervals—Low close monitoring**			
**Standardized direct effects**				**Standardized direct effects**				**Standardized direct effects**			
**Parameter**	**Estimate**	**Lower**	**Upper**	**Parameter**	**Estimate**	**Lower**	**Upper**	**Parameter**	**Estimate**	**Lower**	**Upper**
SL → PE	0.84^**^	0.55	0.99	SL → PE	0.84^**^	0.55	0.99	SL → PE	0.84^**^	0.55	0.99
SL → TProf	0.46	−1.03	2.50	SL → TAdapt	0.23	−1.78	0.93	SL → TPro	0.27	−1.85	0.91
PE → TProf	0.23	−1.87	1.56	PE → TAdapt	0.65	−0.24	2.33	PE → TPro	0.60	−0.14	2.39
**Standardized indirect effects**				**Standardized indirect effects**				**Standardized indirect effects**			
SL → PE → TProf	0.20	−1.23	2.24	SL → PE → TAdapt	0.54^*^	0.10	3.08	SL → PE → TPro	0.51^*^	0.04	4.01
**Direct and indirect effects and 95% confidence intervals—High close monitoring**				**Direct and indirect effects and 95% confidence intervals—High close monitoring**				**Direct and indirect effects and 95% confidence intervals—High close monitoring**			
**Standardized direct effects**				**Standardized direct effects**				**Standardized direct effects**			
**Parameter**	**Estimate**	**Lower**	**Upper**	**Parameter**	**Estimate**	**Lower**	**Upper**	**Parameter**	**Estimate**	**Lower**	**Upper**
SL → PE	0.24	−0.27	0.61	SL → PE	0.24	−0.30	0.61	SL → PE	0.27	−0.30	0.61
SL → TProf	−0.04	−0.65	0.42	SL → TAdapt	0.45	−0.03	0.87	SL → TPro	0.31	0.00	0.79
PE → TProf	0.63^**^	0.18	1.06	PE → TAdapt	0.31	−0.14	0.67	PE → TPro	0.44	−0.04	0.74
**Standardized indirect effects**				**Standardized indirect effects**				**Standardized indirect effects**			
SL → PE → TProf	0.15	−0.20	0.64	SL → PE → TAdapt	0.18	−0.04	0.35	SL → PE → TPro	0.12	−0.04	0.44

*n* = 135 (bootstrapping by specifying a sample of size 5,000). SL, self-leadership; PE, psychological empowerment; TProf, task proficiency; TAdapt, task adaptivity; TPro, task proactivity.

* *p* < 0.05;

** *p* < 0.01.

## Discussion

In this study, we aimed to understand the relationships among self-leadership, psychological empowerment, and work role performance, and scrutinize the moderating effect of supervisor close monitoring on these relationships. Drawing primarily on SDT (Deci et al., 2017), we developed two hypotheses: First, we suggested that psychological empowerment, as an autonomous motivational state, links self-leadership to positive performance outcomes, including increased task proficiency, adaptivity, and proactivity. Second, we considered supervisor close monitoring as an external control and regulation mechanism that exacerbates the previously described self-empowerment process.

We conducted two separate studies to test these hypotheses. In Study 1, we found that psychological empowerment played a mediator role in the relationship between self-leadership and work role performance. This finding was supported by our cross-lagged design in Study 2. Furthermore, in line with Hypothesis 2, we found that supervisor close monitoring moderated the indirect effect of self-leadership on task adaptivity and proactivity such that this effect was non-significant under high supervisor close monitoring. However, we did not obtain the same finding for task proficiency. Hence, Hypothesis 2 was partially supported.

### Theoretical implications

From a theoretical standpoint, our findings extend the boundaries of psychological empowerment theory (Zimmerman, 1995, 2000) and SDT (Deci et al., 2017) to self-leadership research by emphasizing the motivational process of self-empowerment, which places employees at the heart of their own empowerment (Matsuo, 2019; van der Stoep, 2019). In this study, we presented that self-leadership is likely to enhance employees’ psychological empowerment that has spillover effects on subsequent performance outcomes. Our results implied that self-leadership helps employees find their job more meaningful and feel more competent, autonomous, and impactful in their jobs, which in turn contributes to their task proficiency, adaptivity, and proactivity. With these findings, we also addressed an important gap in the current research regarding the empowering mechanisms that connect self-leadership to performance outcomes. Although previous studies have focused on the mediating impact of individual differences and affective-psychological states (i.e., self-efficacy and job satisfaction) in the relationship between self-leadership and employee performance, the literature lacks theoretical and empirical evidence regarding the impact of autonomous motivational states in translating the self-leadership practices into superior work role performance. Drawing upon the basic premises of “workplace self-determination model” (Deci et al., 2017), we attract attention to this “black box” issue of self-empowerment and move beyond the findings of previous studies.

Moreover, our study sheds light on whether and how supervisors’ close monitoring shapes the employees’ self-empowerment process. In line with the previous studies which underlined the drawbacks of close monitoring particularly in traditional work settings (e.g., Son et al., 2017; Kim, 2019; Lee et al., 2019), our results showed that supervisor close monitoring alleviated the indirect positive impact of self-leadership on employees’ task adaptivity and proactivity under remote working conditions. This finding implies that close monitoring by supervisors, which characterizes an external control or regulatory mechanism according to SDT, might damage self-leaders’ autonomous motivation to perform adaptively and proactively. Since both adaptivity and proactivity are change-oriented behaviors and require self-initiated future-oriented actions (Griffin et al., 2010), supervisor close monitoring might interfere with self-leaders’ autonomy and make them conform to the rules and expectations of their supervisors. As a result, these self-leading employees might work just enough to avoid punishment and criticism (Son et al., 2017) and show little or no effort to enhance their adaptive or proactive performance (Rietzschel et al., 2014; Lee et al., 2019).

On the other hand, our findings indicated that self-leadership had no indirect impact on employees’ task proficiency (*via* psychological empowerment) either under high or low close monitoring. The reason for this may be those subordinates, particularly those with self-leading capabilities, need an adequate supervision (i.e., not extremely loose, or tight control; Son et al., 2017) to feel more empowered and show higher performance in remote working settings. This is also an ongoing debate in the teleworking research where some researchers recommend a new way of supervision including more directive behaviors while others do not support the tight control of the tasks (Dimitrova, 2003; Lautsch et al., 2009). Our findings substantiate both views by implying that self-leaders might perceive extremely high levels of external control on regular tasks as excessive supervision and low levels of control as inadequate supervision under remote work conditions. As such, on the one hand, high levels of supervisory control might damage the trust-based supervisor-subordinate relationship and break the spell of psychological empowerment for self-leaders. On the other hand, self-leaders might perceive low levels of supervisory control as negligent behavior (Choi et al., 2009) as they fail to receive clear information and feedback regarding the core aspects of their job in the uncertain, remote working environment (Son et al., 2017). That is, although self-leaders can set their own goals and working toward them, they might still need some coaching or guidance, particularly in remote work settings, regarding performance expectations of their superiors. This is because while working remotely self-leaders might develop concerns about being professionally isolated, namely they might “fear that when they are out of sight, they are out of mind for promotions and other organizational rewards” (Kurland and Cooper, 2002; p. 111).

Taken together, our findings speak to the importance of considering employee needs, perceptions and expectations, work context, and the different aspects of work role performance while evaluating the moderating role of supervisory control in the employee-initiated self-empowerment process.

### Practical implications

Our study has several implications for organizations and managers who aim to enhance their employees’ psychological empowerment and work role performance, particularly within the remote work context. First, managers need to encourage their subordinates to freely use their self-leadership skills as this will increase their autonomous motivation and help them feel more psychologically empowered. In line with Manz (1992), who portrays self-leadership capability as “truly the heart of empowerment” (p. 9), we found that self-leadership enhanced employees’ psychological empowerment, which in turn increased their work role performance. As such, from a human resource perspective, organizations need to lay the necessary groundwork for the development and implementation of self-leadership skills among their existing employees and consider these critical skills as a part of their recruitment and selection efforts as well as their performance evaluation and incentive structures.

Second, our results suggest that self-leadership is a key merit for enhancing employees’ work-role performance particularly in remote work conditions in which individuals need to motivate and regulate themselves in most cases. Hence, it is critical to support employees with ongoing professional trainings that will improve their self-leadership and self-regulation skills. Previous research has validated this argument empirically by showing that individuals who received self-leadership trainings (e.g., thought self-leadership training) experienced increased mental performance, positive affect, job satisfaction and decreased negative affect compared with those who do not receive such trainings (Neck and Manz, 1996). In a recent study, Goldsby et al. (2021) have particularly underlined that self-leadership trainings act as a catalyzer to enhance the individual performance of those who are receiving professional improvement programs. Specifically, the authors have proposed that (certified) professional training programs would be more effective for employees with strong self-leadership skills in that self-leaders would know how to apply the insights of the trainings over time (Goldsby et al., 2021), which would save organizations from the costs of providing similar trainings periodically.

Third, our findings demonstrated that high supervisor close monitoring impaired the psychological empowerment of self-leaders and diminished their willingness to behave adaptively and proactively in their job. Self-leaders may perceive high close monitoring as an intimidation or a pressure for adjusting to the expectations of their supervisors. As a result, these employees may refrain from using novel ways of thinking or finding paths for adaptation. Our findings revealed that, particularly in situations where employee adaptivity and proactivity matter, supervisors should avoid constantly looking over self-leaders’ shoulders and let these employees use their self-management skills. On the other hand, our findings also implied that even though self-leaders have the capacity to self-manage and self-monitor their core tasks (which is represented by task proficiency), they might still need a certain level of supervision (Son et al., 2017). This need might be closely associated with the conditions of constant change and uncertainty in new work environment. Under these circumstances, the level of monitoring that the employees receive from their supervisors is critical as it affects whether and how self-leaders capitalize on close supervision to attain superior work role performance. When the supervisor monitoring is adequate, self-leaders can get sufficient feedback and guidance to nurture their empowerment at work and enhance their core task performance. On the other hand, when the level of supervisor close monitoring is on extremes (very high or very low), it might impair self-leaders’ empowerment and the resulting job performance. Accordingly, supervisors should neither closely monitor nor leave self-leaders completely on their own in the accomplishment of their core tasks.

### Limitations and future research agenda

Our research has some limitations that might guide the future research. The first limitation is the common method bias and the use of a self-reported measure of work role performance in Study 1. Although we intended to overcome this limitation in Study 2 by employing a cross-lagged design and including supervisor-rated employee performance, future research can investigate employees’ work role performance with a much more objective measure. This would better address methodological issues regarding the subjective measurement of performance and the plausible common method bias.^5^

Second, our study investigates the performance outcomes of self-leadership and supervisor close monitoring with samples from Turkey that has been characterized as a power-distant, collectivist country (Hofstede, 1980; Aycan, 2008; Bedi, 2020). Yet, prior research has revealed that even the cross-cultural generalizability of self-leadership dimensions is lacking and thus scholars need to work with cross-cultural samples to identify generalizable self-leadership behaviors (Neubert et al., 2006; Georgianna, 2007). Based on this, future research can gather data from cross-culturally comparative samples or within different cultural contexts to validate our findings and to investigate whether and how cultural values or characteristics moderate the performance outcomes of self-leadership.

Another limitation of our study lies in the small sample size of both studies. Although we endeavored to increase the sample size particularly in the second study, conducting the data collection in two different waves and receiving performance evaluations from direct supervisors made it difficult to increase the sample size within the predetermined time frame and budget of the project. On the other hand, despite the small sample size and using a time-lagged design, we were able to validate most of our hypotheses. Still, future studies might replicate and extend the current findings with larger and more representative samples, in which the respondents work remotely for extended time periods.

In our study, we aimed to reach employees who worked primarily from home and who had minimum interaction with their supervisors and coworkers in the office environment. Although the majority of the employees in our sample were full-time remote workers (n_full-time_ = 146 out of 174, 84% for Study 1 and n_full-time_ = 146 out of 160, 91%; n_full-time_ = 126 out of 135, 93% for Study 2, Time 1 and Time 2, respectively), we acknowledge the need for future studies to test our model with employees who work as permanent, full-time remote workers.

Finally, our findings suggest that the outcomes of close supervision for self-leaders may heavily depend on the level of monitoring is performed by supervisors as well as the type of expected performance outcome. In the case of adaptive and proactive performance, self-leaders may suffer from close supervision as it might get in the way of their psychological empowerment. On the other hand, supervisor close monitoring, if implemented at an optimum level (i.e., adequate supervision) might have nourishing effects on self-leaders’ core job performance. Hence, it is important for future studies to clarify what should be the adequate level of monitoring that is implemented by the supervisors for different performance outcomes (i.e., proficiency, adaptivity, proactivity) and in in different work contexts (e.g., remote work, virtual teams).

## Conclusion

Exploring the indirect impact of self-leadership on work role performance *via* psychological empowerment, this study revealed that psychological empowerment is an influential mechanism that links self-leadership to work role performance. On the other hand, the moderating impact of supervisor close monitoring was found for two of the work role performance outcomes (task adaptivity and proactivity). Such findings are noteworthy for managers and human resource management professionals as they speak to the importance of (a) laying the necessary ground for the development and implementation of self-leadership skills in remote work settings and (b) determining the appropriate level of monitoring provided to self-leaders—as it might be necessary to enhance their task proficiency, but redundant for increasing their task adaptivity and proactivity.

## Data availability statement

The raw data supporting the conclusions of this article will be made available by the authors, without undue reservation.

## Ethics statement

Ethical review and approval was not required for the study on human participants in accordance with the local legislation and institutional requirements. The patients/participants provided their written informed consent to participate in this study.

## Author contributions

All authors listed have made a substantial, direct, and intellectual contribution to the work and approved it for publication.

## Conflict of interest

The authors declare that the research was conducted in the absence of any commercial or financial relationships that could be construed as a potential conflict of interest.

## Publisher’s note

All claims expressed in this article are solely those of the authors and do not necessarily represent those of their affiliated organizations, or those of the publisher, the editors and the reviewers. Any product that may be evaluated in this article, or claim that may be made by its manufacturer, is not guaranteed or endorsed by the publisher.

## Footnotes

^1^The first COVID-19 case in Turkey was reported on 11 March 2020, and initial precautions were taken subsequently. Namely, thousands of people were quarantined, and schools, universities, and business enterprises were locked down. With the country’s infection rate among the highest in Europe, in April 2021, Turkey entered its first nationwide lockdown (BBC News, 2021). In the initial phases, Turkey seemed to have coped relatively well with the pandemic considering the low number of confirmed cases, timely isolation, protection, and tracing measures, and strong healthcare system (e.g., The Turkish health system has the highest number of intensive care units in the world at 46.5 beds per 100,000 people; Aydın-Düzgit et al., 2021; DBA Turkey, 2021). In the later stages, however, certain administrative and capacity-related problems arose. By the end of August 2021, Turkey had the higher cumulative number of positive cases (per million people) than many other countries (Our World in Data, 2022). The government initiated a massive vaccination campaign in January 2021, primarily covering healthcare workers and elderly citizens. Although the government kept many businesses open and allowed companies to determine their own guidelines regarding workers throughout the pandemic (Economist, 2020), private enterprises were strongly encouraged to switch to remote work at different phases of the pandemic.

^2^In all CFAs, psychological empowerment was included as higher-order construct because the higher-order model demonstrated a significantly better fit than the single-factor model [∆χ2(4) = 700.776, *p* < 0.01] and the orthogonal first-order model (i.e., the model in which the four lower-order factors are uncorrelated; ∆χ2(4) = 183.908, *p* < 0.01). The higher-order model exhibited a similar fit to the oblique first-order model in which the four lower-order factors are correlated [∆χ2(2) = 3.32, *p* < 0.01]. Credé and Harms (2015) suggested that the validity of higher-order model should not be presumed as a result of only model comparisons and researchers should apply further tests. In this study, we apply the "Target Coefficient (TC) 1" and "Target Coefficient 2" tests (Marsh, 1987) to evaluate whether the higher-order factor of empowerment sufficiently explained the covariation among the first-order factors. Both the TC1 (0.96) and the TC2 (0.96) were close to 1 which showed support for the higher order modeling (Marsh, 1987).

^3^Model comparisons for Time 1 and Time 2 data can be found at Appendix Table A2.

^4^Because the samples in multi-group analysis consist of only 66 (for low supervisor close monitoring) and 69 (for high supervisor close monitoring) employees, there was concern about statistical power of the analysis. Thus, a *post-hoc* power analysis was performed (Cohen, 1988) for the non-significant indirect effect of self-leadership on task proficiency using the online tool created by Soper (2020). For both groups (i.e., low and high supervisor close monitoring), observed statistical power of the non-significant effect was 0.99 at the 0.05 level (*R^2^_low_* = 0.55, *R^2^_high_* = 0.39), which was above the threshold of 0.8. As such, adequate statistical power was achieved for the non-significant indirect effect in question.

^5^To deal with the common method bias in both studies, we applied some procedural remedies. In the survey design, items of the mediator variable (psychological empowerment) were presented before the independent variable (self-leadership) as recommended by Podsakoff et al. (2003). Moreover, we measured all variables using well-validated scales to reduce the measurement error and thereby to minimize the occurrence of common method bias. Finally, in Study 1, we measured employees’ job performance 1 week after the initial application of the survey.

## References

[ref1] Amundsen S. Martinsen Ø. L. (2015). Linking empowering leadership to job satisfaction, work effort, and creativity: the role of self-leadership and psychological empowerment. J. Leadersh. Org. Stud. 22, 304–323. doi: 10.1177/1548051814565819

[ref2] Avey J. B. Nimnicht J. L. Pigeon N. G. (2010). Two field studies examining the association between positive psychological capital and employee performance. Leadersh. Org. Dev. J. 31, 384–401. doi: 10.1108/01437731011056425

[ref3] Aycan Z. (2008). “Cross-cultural approaches to leadership,” in The handbook of cross cultural management research. eds. Smith P. B. P. Peterson M. F. Thomas D. C. (Thousand Oaks, CA: Sage), 219–238.

[ref4] Aydın-Düzgit S. Kutlay M. Keyman E. F. (2021). Politics of pandemic management in Turkey. IPC Policy Brief, 20211025–00101541.

[ref5] Bailey S. F. Barber L. K. Justice L. M. (2018). Is self-leadership just self-regulation? Exploring construct validity with HEXACO and self-regulatory traits. Curr. Psychol. 37, 149–161. doi: 10.1007/s12144-016-9498-z

[ref6] BBC News (2021). Covid: Turkey enters first full lockdown (29 April 2021). Available at: https://www.bbc.com/news/world-europe-56912668 (Accessed October 9, 2022).

[ref7] Bedi A. (2020). A meta-analytic review of paternalistic leadership. App. Psy. 69, 960–1008. doi: 10.1111/apps.12186

[ref8] Bindl U. K. Parker S. K. (2010). “Proactive work behavior: forward-thinking and change-oriented action in organizations,” in APA handbook of industrial and organizational psychology. *Vol*. 2. ed. Zedeck S. (Washington, DC: American Psychological Association), 567–598.

[ref9] Bonias D. Bartram T. Leggat S. G. Stanton P. (2010). Does psychological empowerment mediate the relationship between high performance work systems and patient care quality in hospitals? Asia Pac. J. Hum. Resour. 48, 319–337. doi: 10.1177/1038411110381667

[ref10] Brislin R. W. (1986). “The wording and translation of research instruments,” in Field methods in cross-cultural research. eds. Lonner W. J. Berry J. W. (Beverly Hills, CA: Sage), 137–164.

[ref11] Castellano S. Chandavimol K. Khelladi I. Orhan M. A. (2021). Impact of self-leadership and shared leadership on the performance of virtual R & D teams. J. Bus. Res. 128, 578–586. doi: 10.1016/j.jbusres.2020.12.030

[ref12] Cerasoli C. P. Nicklin J. M. Ford M. T. (2014). Intrinsic motivation and extrinsic incentives jointly predict performance: a 40-year meta-analysis. Psychol. Bull. 140, 980–1008. doi: 10.1037/a0035661, PMID: 24491020

[ref13] Chaijukul Y. (2010). An examination of self-leadership performance mechanism model in Thai private organization. J. Behav. Sci. 5, 1–14. doi: 10.1007/s12144-021-01697-5, PMID: 33867780 PMC8043442

[ref14] Choi J. N. Anderson T. A. Veillette A. (2009). Contextual inhibitors of employee creativity in organizations: the insulating role of creative ability. Gr. Organ. Manag. 34, 330–357. doi: 10.1177/1059601108329811

[ref15] Chong S. Huang Y. Chang C. H. D. (2020). Supporting interdependent telework employees: a moderated-mediation model linking daily COVID-19 task setbacks to next-day work withdrawal. J. Appl. Psychol. 105, 1408–1422. doi: 10.1037/apl0000843, PMID: 33271029

[ref16] Cohen J. (1988). Statistical power analysis for the behavioral sciences. 2nd *Edn*. Hillsdale, New Jerssey, NJ: Lawrence Earlbaum Associates.

[ref17] Costantini A. Weintraub J. (2022). The benefits of being proactive while working remotely: leveraging self-leadership and job crafting to achieve higher work engagement and task significance. Front. Psychol. 13:833776. doi: 10.3389/fpsyg.2022.833776, PMID: 35548485 PMC9082026

[ref001] Credé M. Harms P. D. (2015). 25 years of higher-order confirmatory factor analysis in the organizational sciences: a critical review and development of reporting recommendations. J. of Organ. Behav. 36, 845–872. doi: 10.1002/job.2008

[ref18] DBA Turkey. (2021). Covid-19 situation in Turkey. Available at: https://www.dbaturkey.org/covid-19/ (Accessed October 9, 2022).

[ref19] Deci E. L. Olafsen A. H. Ryan R. M. (2017). Self-determination theory in work organizations: the state of a science. Ann. Rev. Organ. Psychol. Organ. Behav. 4, 19–43. doi: 10.1146/annurev-orgpsych-032516-113108

[ref20] Dimitrova D. (2003). Controlling teleworkers: supervision and flexibility revisited. New Technol. Work Employ. 18, 181–195. doi: 10.1111/1468-005X.00120

[ref21] Economist (2020). What Turkey got right about the pandemic. Available at: https://www.economist.com/europe/2020/06/04/what-turkey-got-right-about-the-pandemic (Accessed October 9, 2022).

[ref22] Forbes (2021). Monitoring remote workers: The good, the bad and the ugly. Available at: https://www.forbes.com/sites/forbesagencycouncil/2021/12/08/monitoring-remote-workers-the-good-the-bad-and-the-ugly/?sh=77d7b0b11da8 (Accessed October 11).

[ref23] Fornell C. Larcker D. F. (1981). Evaluating structural equation models with unobservable variables and measurement error. J. Market. Res. 18, 39–50. doi: 10.1177/002224378101800104

[ref24] Gan J. Zhou Z. E. Tang H. Ma H. Gan Z. (2022). What it takes to be an effective “remote leader” during covid-19 crisis: the combined effects of supervisor control and support behaviors. Int. J. Hum. Res. Man. doi: 10.1080/09585192.2022.2079953

[ref25] García-Chas R. Neira-Fontela E. Varela-Neira C. (2015). Comparing the explanatory capacity of three constructs in the prediction of engineers’ proficiency, adaptivity, and proactivity. Hum. Resour. Manage. 54, 689–709. doi: 10.1002/hrm.21639

[ref26] George J. M. Zhou J. (2001). When openness to experience and conscientiousness are related to creative behavior: an interactional approach. J. Appl. Psychol. 86, 513–524. doi: 10.1037/0021-9010.86.3.513, PMID: 11419810

[ref27] Georgianna S. (2007). Self-leadership: a cross-cultural perspective. J. Man. Psy. 22, 569–589. doi: 10.1108/02683940710778440

[ref28] Gephart R. P. Jr. (2002). Introduction to the brave new workplace: organizational behavior in the electronic age. J. Organ. Behav. 23, 327–344. doi: 10.1002/job.143

[ref29] Goldsby M. G. Goldsby E. A. Neck C. B. Neck C. P. Mathews R. (2021). Self-leadership: a four-decade review of the literature and trainings. Admin. Sci. 11:25. doi: 10.3390/admsci11010025

[ref30] Gong B. Sims R. L. (2023). Psychological contract breach during the pandemic: how an abrupt transition to a work from home schedule impacted the employment relationship. J. Bus. Res. 154:113259. doi: 10.1016/j.jbusres.2022.08.023, PMID: 36089927 PMC9448651

[ref31] Griffin M. A. Neal A. Parker S. K. (2007). A new model of work role performance: positive behavior in uncertain and interdependent contexts. Acad. Manage. J. 50, 327–347. doi: 10.5465/amj.2007.24634438

[ref002] Grant A. M. Parker S. K. (2009). Redesigning work design theories: the rise of relational and proactive perspectives. Acad. of Manage. Ann. 317–375. doi: 10.1080/19416520903047327, PMID: 36089927

[ref32] Griffin M. A. Parker S. K. Mason C. M. (2010). Leader vision and the development of adaptive and proactive performance: a longitudinal study. J. Appl. Psychol. 95, 174–182. doi: 10.1037/a0017263, PMID: 20085414

[ref33] Hauschildt K. Konradt U. (2012). Self-leadership and team members' work role performance. J. Manag. Psychol. 27, 497–517. doi: 10.1108/02683941211235409

[ref34] Henseler J. Ringle C. M. Sarstedt M. (2015). A new criterion for assessing discriminant validity in variance-based structural equation modeling. J. Acad. Market Sci. 43, 115–135. doi: 10.1007/s11747-014-0403-8

[ref35] Hofstede G. (1980). Culture’s consequences: International differences in work-related values. Beverly Hills, CA: Sage Publications.

[ref36] Houghton J. D. Dawley D. DiLiello T. C. (2012). The abbreviated self-leadership questionnaire (ASLQ): a more concise measure of self-leadership. Internat. J. Leadersh. Stud. 7, 216–232.

[ref37] Houghton J. D. Neck C. P. (2002). The revised self-leadership questionnaire: testing a hierarchical factor structure for self-leadership. J. Man. Psychol. 17, 672–691. doi: 10.1108/02683940210450484

[ref38] Houghton J. D. Yoho S. K. (2005). Toward a contingency model of leadership and psychological empowerment: when should self-leadership be encouraged? J. Leadersh. Organ. Stud. 11, 65–83. doi: 10.1177/107179190501100406

[ref39] Hu L. T. Bentler P. M. (1999). Cutoff criteria for fit indexes in covariance structure analysis: conventional criteria versus new alternatives. Struct. Equ. Model. Multidiscip. J. 6, 1–55. doi: 10.1080/10705519909540118

[ref40] Inam A. Ho J. A. Sheikh A. A. Shafqat M. Najam U. (2021). How self-leadership enhances normative commitment and work performance by engaging people at work? Curr. Psychol. 1-14, 1–14. doi: 10.1007/s12144-021-01697-5, PMID: 33867780 PMC8043442

[ref41] Kalra A. Agnihotri R. Singh R. Puri S. Kumar N. (2021). Assessing the drivers and outcomes of behavioral self-leadership. Eur. J. Mark. 55, 1227–1257. doi: 10.1108/EJM-11-2018-0769

[ref42] Kim S. L. (2019). The interaction effects of proactive personality and empowering leadership and close monitoring behaviour on creativity. Creat. Innov. Manag. 28, 230–239. doi: 10.1111/caim.12304

[ref43] Kniffin K. M. Narayanan J. Anseel F. Antonakis J. Ashford S. P. Bakker A. B.. (2021). COVID-19 and the workplace: implications, issues, and insights for future research and action. Am. Psychol. 76, 63–77. doi: 10.1037/amp0000716, PMID: 32772537

[ref44] Konradt U. Andreßen P. Ellwart T. (2009). Self-leadership in organizational teams: a multilevel analysis of moderators and mediators. Eur. J. Work Organ. Psy. 18, 322–346. doi: 10.1080/13594320701693225

[ref45] Kurland N. B. Cooper C. D. (2002). Manager control and employee isolation in telecommuting environments. J. High Technol. Managem. Res. 13, 107–126. doi: 10.1016/S1047-8310(01)00051-7

[ref46] Lautsch B. A. Kossek E. E. Eaton S. C. (2009). Supervisory approaches and paradoxes in managing telecommuting implementation. Hum. Relat. 62, 795–827. doi: 10.1177/0018726709104543

[ref47] Lebel R. D. Patil S. V. (2018). Proactivity despite discouraging supervisors: the powerful role of prosocial motivation. J. Appl. Psychol. 103, 724–737. doi: 10.1037/apl0000301, PMID: 29578739

[ref48] Lee J. Yun S. Lee S. Lee J. (2019). The curvilinear relationship between self-efficacy and creativity: the moderating role of supervisor close monitoring. J. Bus. Psychol. 34, 377–388. doi: 10.1080/713769649

[ref49] Mahembe B. Engelbrecht A. Wakelin Z. (2017). A study to assess the reliability and construct validity of the abbreviated self-leadership questionnaire: a south African study. South Afr. J. Psychol. 47, 356–366. doi: 10.1177/0081246316675139

[ref50] Manz C. C. (1986). Self-leadership: toward an expanded theory of self-influence processes in organizations. Acad. Manage. Rev. 11, 585–600. doi: 10.5465/amr.1986.4306232

[ref51] Manz C. C. (1992). Self-leadership. The heart of empowerment. J. Qual. Part. 15, 80–85.

[ref52] Manz C. C. Sims H. P. Jr. (1980). Self-management as a substitute for leadership: a social learning perspective. Acad. Manage. Rev. 5, 361–367. doi: 10.5465/amr.1980.4288845

[ref53] Marques-Quinteiro P. Curral L. A. (2012). Goal orientation and work role performance: predicting adaptive and proactive work role performance through self-leadership strategies. J. Psychol. 146, 559–577. doi: 10.1080/00223980.2012.656157, PMID: 23094471

[ref54] Marques-Quinteiro P. Vargas R. Eifler N. Curral L. (2019). Employee adaptive performance and job satisfaction during organizational crisis: the role of self-leadership. Eur. J. Work Organ. Psy. 28, 85–100. doi: 10.1080/1359432X.2018.1551882

[ref55] Marsh H. W. (1987). The hierarchical structure of self-concept and the application of hierarchical confirmatory factor analysis. J. Educ. Meas. 24, 17–39. doi: 10.1111/j.1745-3984.1987.tb00259.x

[ref56] Martin S. L. Liao H. Campbell E. M. (2013). Directive versus empowering leadership: a field experiment comparing impacts on task proficiency and proactivity. Acad. Manage. J. 56, 1372–1395. doi: 10.5465/amj.2011.0113

[ref57] Matsuo M. (2019). Personal growth initiative as a predictor of psychological empowerment: the mediating role of job crafting. Hum. Resour. Dev. Quart. 30, 343–360. doi: 10.1002/hrdq.21347

[ref58] Mayfield M. Mayfield J. (2021). Sound and safe: the role of leader motivating language and follower self-leadership in feelings of psychological safety. Admin. Sci. 11, 1–30. doi: 10.3390/admsci11020051

[ref59] Mayfield J. Mayfield M. Neck C. P. (2021). Speaking to the self: how motivating language links with self-leadership. Int. J. Bus. Commun. 58, 31–54. doi: 10.1177/232948841773186

[ref60] Mishra M. Ghosh K. (2020). Supervisor monitoring and subordinate work attitudes: a need satisfaction and supervisory support perspective. Leadersh. Org. Dev. J. 41, 1089–1105. doi: 10.1108/LODJ-05-2019-0204

[ref61] Morin A. J. Meyer J. P. Bélanger É. Boudrias J. S. Gagné M. Parker P. D. (2016). Longitudinal associations between employees’ beliefs about the quality of the change management process, affective commitment to change and psychological empowerment. Hum. Relat. 69, 839–867. doi: 10.1177/0018726715602046

[ref62] Mulaik S. A. James L. R. Van Alstine J. Bennett N. Lind S. Stilwell C. D. (1989). Evaluation of goodness-of-fit indices for structural equation models. Psychol. Bull. 105, 430–445. doi: 10.1037/0033-2909.105.3.430

[ref63] Müller T. Niessen C. (2019). Self-leadership in the context of part-time teleworking. J. Organ. Behav. 40, 883–898. doi: 10.1002/job.2371

[ref64] Neck C. P. Houghton J. D. (2006). Two decades of self-leadership theory and research: past developments, present trends, and future possibilities. J. Manag. Psychol. 21, 270–295. doi: 10.1177/0018726715602046

[ref65] Neck C. P. Manz C. C. (1996). Thought self-leadership: the impact of mental strategies training on employee cognition, behavior, and affect. J. Organ. Behav. 17, 445–467. doi: 10.1002/(SICI)1099-1379(199609)17:5<445::AID-JOB770>3.0.CO;2-N

[ref66] Nel P. Van Zyl E. (2015). Assessing the psychometric properties of the revised and abbreviated self-leadership questionnaires. SA Hum. Resour. Manag. 13, 1–8. doi: 10.4102/sajhrm.v13i1.661

[ref67] Neubert M. J. Cindy W. (2006). Investigation of the generalizability of the Houghton and Neck revised self-leadership questionnaire to a Chinese context. J. Man. Psy. 21, 360–373. doi: 10.1108/02683940610663132

[ref68] Our World in Data (2022). Turkey: Coronavirus pandemic country profile. Available at: https://ourworldindata.org/coronavirus/country/turkey (Accessed October 9, 2022).

[ref69] Panagopoulos N. G. Ogilvie J. (2015). Can salespeople lead themselves? Thought self-leadership strategies and their influence on sales performance. Ind. Mark. Manag. 47, 190–203. doi: 10.1016/j.indmarman.2015.02.043

[ref70] Podsakoff P. M. MacKenzie S. B. Lee J. Y. Podsakoff N. P. (2003). Common method biases in behavioral research: a critical review of the literature and recommended remedies. J. Appl. Psychol. 88, 879–903. doi: 10.1037/0021-9010.88.5.879, PMID: 14516251

[ref71] Rietzschel E. F. Slijkhuis M. Van Yperen N. W. (2014). Close monitoring as a contextual stimulator: how need for structure affects the relation between close monitoring and work outcomes. Eur. J. Work Organ. Psy. 23, 394–404. doi: 10.1080/1359432X.2012.752897

[ref72] Selig J. P. Preacher K. J. (2009). Mediation models for longitudinal data in developmental research. Res. Hum. Dev. 6, 144–164. doi: 10.1080/15427600902911247

[ref73] Sitkin S. B. Roth N. L. (1993). Explaining the limited effectiveness of legalistic “remedies” for trust/distrust. Org. Sci. 4, 367–392. doi: 10.1287/orsc.4.3.367

[ref74] Son S. C. Y. Cho D. H. Kang S. W. (2017). The impact of close monitoring on creativity and knowledge sharing: the mediating role of leader-member exchange. Creat. Innov. Manag. 26, 256–265. doi: 10.1111/caim.12219

[ref75] Soper D. S. (2020). Post-hoc statistical power calculator for multiple regression [software], Available online at: https://www.danielsoper.com/statcalc/ (Accessed October 10, 2022).

[ref76] Spreitzer G. M. (1995). Psychological empowerment in the workplace: dimensions, measurement, and validation. Acad. Manage. J. 38, 1442–1465. doi: 10.5465/256865

[ref77] Sun L. Y. Zhang Z. Qi J. Chen Z. X. (2012). Empowerment and creativity: a cross-level investigation. Leadersh. Q. 23, 55–65. doi: 10.1016/j.leaqua.2011.11.005

[ref78] Thomas K. W. Velthouse B. A. (1990). Cognitive elements of empowerment: an “interpretive” model of intrinsic task motivation. Acad. Manage. J. 15, 666–681. doi: 10.5465/amr.1990.4310926

[ref79] Thomas J. P. Whitman D. S. Viswesvaran C. (2010). Employee proactivity in organizations: a comparative meta-analysis of emergent proactive constructs. J. Occup. Organ. Psychol. 83, 275–300. doi: 10.1348/096317910X502359

[ref80] van der Stoep J. J. (2019). Power to the people: employee empowerment in contemporary organizations. Available at: https://research.vu.nl/ws/portalfiles/portal/79047696/complete+dissertation.pdf

[ref81] Vandenberg R. J. Lance C. E. (2000). A review and synthesis of the measurement invariance literature: suggestions, practices, and recommendations for organizational research. Organ. Res. Meth. 3, 4–70. doi: 10.1177/109442810031002

[ref82] Wang B. Liu Y. Qian J. Parker S. K. (2021). Achieving effective remote working during the COVID-19 pandemic: a work design perspective. App. Psychol. 70, 16–59. doi: 10.1111/apps.12290, PMID: 33230359 PMC7675760

[ref83] Whitener E. M. Brodt S. E. Korsgaard M. A. Werner J. M. (1998). Managers as initiators of trust: an exchange relationship framework for understanding managerial trustworthy behavior. Acad. Manage. Rev. 23, 513–530. doi: 10.2307/259292

[ref84] Wilson J. H. (2011). Freedom at work: psychological empowerment and self-leadership. Inter. J. Bus. Pub. Admin. 8, 106–124.

[ref003] Williams L. J. Anderson S. E. (1991). Job satisfaction and organizational commitment as predictors of organizational citizenship and in-role behaviors. J. of Manage. 17, 601–617. doi: 10.1177/014920639101700305, PMID: 35783713

[ref85] Wu C.-H. Tian A. W. Luksyte A. Spitzmueller C. (2017). On the association between perceived overqualification and adaptive behavior. Pers. Rev. 46, 339–354. doi: 10.1108/PR-05-2015-0134

[ref86] Xu Y. Zhang M. (2022). The study of the impact of empowering leadership on adaptive performance of faculties based on chain mediating. Front. Psychol. 13:938951. doi: 10.3389/fpsyg.2022.938951, PMID: 35783713 PMC9247655

[ref87] Zhang M. J. Law K. S. Lin B. (2016). You think you are big fish in a small pond? Perceived overqualification, goal orientations, and proactivity at work. J. Organ. Behav. 37, 61–84. doi: 10.1002/job.2024

[ref88] Zhang X. Qian J. Wang B. Jin Z. Wang J. Wang Y. (2017). Leaders’ behaviors matter: the role of delegation in promoting employees’ feedback-seeking behavior. Front. Psychol. 8:920. doi: 10.3389/fpsyg.2017.00920, PMID: 28638357 PMC5461250

[ref89] Zhou J. (2003). When the presence of creative coworkers is related to creativity: role of supervisor close monitoring, developmental feedback, and creative personality. J. Appl. Psychol. 88, 413–422. doi: 10.1037/0021-9010.88.3.413, PMID: 12814291

[ref004] Zimmerman M. A. (1995). Psychological empowerment: Issues and illustrations. Amer. J. of Commun. Psychol. 23, 581–599. doi: 10.1007/BF02506983 8851341

[ref005] Zimmerman M. A. (2000). “Empowerment theory: Psychological, organizational, and community levels of analysis,” in Handbook of Community Psychology. eds. Rappaport Seidman E. (New York, NY: Kluwer Academic/Plenum), 43–63.

