# Supplementary material for: Captain or deckhand? The impact of self-leadership on employees’ work role performance under remote work
Source: Front Psychol. 2022 Nov 25;13:988105. doi: 10.3389/fpsyg.2022.988105 (PMC9732464; doi:10.3389/fpsyg.2022.988105)
Supplement: Supplementary file 1 [file Data_Sheet_1.docx]

**APPENDIX**

Supervisor Close Monitoring Scale (George and Zhou, 2001)

1. It sometimes feels like my supervisor is always looking over my shoulder.
2. I am careful not to do things that my supervisor might disapprove of.
3. My supervisor keeps pretty close tabs on me.
4. It is clear to me that to get ahead in this organization, I need to do exactly what I am told.
5. My supervisor likes to see things done in a certain way.
6. My work is constantly being evaluated.

**TABLE A1** Measurement model comparisons (Study 1).

| Models | Factors | χ2 | df | Δχ2 | CFI | TLI | RMSEA | SRMR |
| --- | --- | --- | --- | --- | --- | --- | --- | --- |
| Full measurement model^a^ | Self-leadership, PE, Task Proficiency, Task Adaptivity, Task Proactivity | 340.86 | 197 | - | 0.92 | 0.91 | 0.07 | 0.07 |
| Four factors model | Combined Self-leadership and PE into one factor | 468.28 | 201 | 127.42** | 0.86 | 0.84 | 0.09 | 0.08 |
| Three factors model | Combined Self-leadership and PE into one factor + Combined Task Proficiency and Task Adaptivity into another factor | 585.72 | 204 | 244.86** | 0.80 | 0.71 | 0.10 | 0.09 |
| Three factors model | Combined Self-leadership and PE into one factor + Combined Task Proficiency and Task Proactivity into another factor | 603.06 | 204 | 262.20** | 0.75 | 0.72 | 0.12 | 0.09 |
| Three factors model | Combined Self-leadership and PE into one factor + Combined Task Adaptivity and Task Proactivity into another factor | 539.23 | 204 | 198.37** | 0.82 | 0.80 | 0.10 | 0.09 |
| Two factors model | Combined Self-leadership and PE into one factor + Combined Task Proficiency, Task Adaptivity and Task Proactivity into another factor | 696.39 | 206 | 355.53** | 0.74 | 0.71 | 0.12 | 0.10 |
| Single factor model | Combined Self-leadership, PE, Task Proficiency, Task Adaptivity and Task Proactivity into one factor | 893.18 | 207 | 552.32** | 0.63 | 0.59 | 0.14 | 0.12 |
| Two factors model (Measurement model without supervisor-rated task proficiency, task adaptivity, and task proactivity) | Self-leadership and PE | 121.95 | 62 | - | 0.93 | 0.91 | 0.08 | 0.07 |
| Single factor model | Combined Self-leadership and PE | 398.05 | 65 | 276.1** | 0.59 | 0.51 | 0.17 | 0.12 |
| Three factors model (Two factors model + single method factor) | Self-leadership, PE + ingle method factor | 162.55 | 51 | 40.6** | 0.86 | 0.79 | 0.11 | 0.07 |

Notes: n= 174, **p<0.01; a= Five factors model; *χ2*, chi-square discrepancy; *df*, degrees of freedom; Δχ2, difference in chi-square; CFI, comparative fit index; TLI, Tucker Lewis index; RMSEA, root-mean square error of approximation; SRMR, standardized root mean square residual. PE= Psychological empowerment

| Models | Factors | χ2 | df | Δχ2 | CFI | TLI | RMSEA | SRMR |
| --- | --- | --- | --- | --- | --- | --- | --- | --- |
| Time 1 |  |  |  |  |  |  |  |  |
| Three factors model | Self-leadership, PE, Supervisor close monitoring | 168.47 | 101 | - | 0.94 | 0.93 | 0.07 | 0.05 |
| Two factors model | Combined Self-leadership and PE into one factor | 210.01 | 103 | 41.54** | 0.93 | 0.92 | 0.06 | 0.06 |
| Two factors model | Combined Self-leadership and Supervisor close monitoring into one factor | 362.23 | 103 | 193.76** | 0.78 | 0.74 | 0.13 | 0.10 |
| Two factors model | Combined PE and Supervisor close monitoring into one factor | 382.75 | 103 | 214.28** | 0.81 | 0.78 | 0.12 | 0.09 |
| Single factor model | Combined Self-leadership, PE, Supervisor close monitoring into one factor | 384.96 | 104 | 216.49** | 0.76 | 0.72 | 0.13 | 0.11 |
| Three factors model + single method factor | Self-leadership, PE, Supervisor close monitoring + single method factor | 188.11 | 90 | 19.63** | 0.92 | 0.89 | 0.08 | 0.06 |
| Time 2 |  |  |  |  |  |  |  |  |
| Two factors model | Self-leadership and PE | 68.10 | 34 | - | 0.94 | 0.92 | 0.09 | 0.05 |
| Single factor model | Combined Self-leadership and PE | 117.00 | 35 | 48.90** | 0.87 | 0.83 | 0.13 | 0.07 |
| Two factors model+ single method factor | Self-leadership and PE + single method factor | 63.30 | 24 | 4.8 | 0.93 | 0.87 | 0.11 | 0.06 |

**TABLE A2** Measurement model comparisons (Study 2 – Time 1 and Time 2).

Notes: n= 160 for T1 and 135 for T2, **p<0.01; *χ2*, chi-square discrepancy; *df*, degrees of freedom; Δχ2, difference in chi-square; CFI, comparative fit index; TLI, Tucker Lewis index; RMSEA, root-mean square error of approximation; SRMR, standardized root mean square residual. PE= Psychological empowerment

| **Variable** | **min** | **Max** | **skew** | **kurtosis** |
| --- | --- | --- | --- | --- |
| **Gender** | .00 | 1.00 | .423 | -1.821 |
| **WorkExp** | 1.00 | 39.00 | .403 | -.022 |
| **Tenure** | 1.00 | 31.00 | .782 | .072 |
| **TaskPro3** | 1.00 | 5.00 | -.296 | .008 |
| **TaskPro2** | 1.00 | 5.00 | -.531 | -.235 |
| **TaskPro1** | 1.00 | 5.00 | -.570 | .045 |
| **TaskAdap3** | 1.00 | 5.00 | -.659 | .099 |
| **TaskAdap2** | 1.00 | 5.00 | -.805 | .653 |
| **TaskAdap1** | 1.00 | 5.00 | -1.088 | 1.954 |
| **TaskProf3** | 1.00 | 5.00 | -1.815 | 4.512 |
| **TaskProf2** | 1.00 | 5.00 | -1.617 | 4.200 |
| **TaskProf1** | 1.00 | 5.00 | -2.152 | 5.935 |
| **Self-Lead1** | 1.00 | 5.00 | -.490 | -.300 |
| **Self-Lead2** | 1.00 | 5.00 | -.670 | .048 |
| **Self-Lead3** | 1.00 | 5.00 | -.911 | -.138 |
| **Self-Lead4** | 1.00 | 5.00 | -1.026 | .789 |
| **Self-Lead5** | 1.00 | 5.00 | -.736 | .036 |
| **Self-Lead6** | 1.00 | 5.00 | -.873 | .544 |
| **Self-Lead7** | 1.00 | 5.00 | -.794 | 1.290 |
| **Self-Lead8** | 1.00 | 5.00 | -.921 | 2.151 |
| **Self-Lead9** | 1.00 | 5.00 | -.890 | 1.474 |
| **PempImp** | 1.00 | 5.00 | -.478 | -.324 |
| **PempSD** | 1.00 | 5.00 | -.799 | 1.014 |
| **PempComp** | 1.00 | 5.00 | -1.901 | 7.835 |
| **PempMean** | 1.00 | 5.00 | -1.318 | 2.305 |

**TABLE A3** Assessment of Normality – Study 1 (Skewness and Kurtosis values).

**TABLE A4** Assessment of Normality – Study 2 (Skewness and Kurtosis values).

|  | **Min** | **max** | **skew** | **kurtosis** |
| --- | --- | --- | --- | --- |
| **Gender** | .00 | 1.00 | .015 | -2.00 |
| **WorkExp** | .500 | 27.00 | .733 | -.300 |
| **Tenure** | .160 | 25.00 | 2.852 | 14.455 |
| **ImpactT1** | 1.00 | 5.00 | -.383 | -.374 |
| **SelfDT1** | 1.330 | 5.00 | -.353 | -.946 |
| **CompT1** | 1.00 | 5.00 | -.561 | -.870 |
| **MeanT1** | 1.330 | 5.00 | -.556 | -.543 |
| **SelfLead1T1** | 1.00 | 5.00 | -.451 | -.371 |
| **SelfLead2T1** | 1.00 | 5.00 | -.150 | -.587 |
| **SelfLead3T1** | 1.00 | 5.00 | -.330 | -.695 |
| **SelfLead4T1** | 1.00 | 5.00 | -.340 | -.842 |
| **SelfLead5T1** | 1.00 | 5.00 | .031 | -.553 |
| **SelfLead6T1** | 1.00 | 5.00 | -.339 | -.704 |
| **SelfLead1T2** | 1.00 | 5.00 | -.438 | .131 |
| **SelfLead2T2** | 1.00 | 5.00 | -.365 | -.128 |
| **SelfLead3T2** | 1.00 | 5.00 | -.243 | -.229 |
| **SelfLead4T2** | 1.00 | 5.00 | -.170 | -.387 |
| **SelfLead5T2** | 1.00 | 5.00 | -.133 | -.195 |
| **SelfLead6T2** | 1.00 | 5.00 | -.492 | .429 |
| **SupCont6** | 1.00 | 5.00 | -.265 | -.512 |
| **SupCont5** | 1.00 | 5.00 | -.285 | -.683 |
| **SupCont4** | 1.00 | 5.00 | -.286 | -.480 |
| **SupCont3** | 1.00 | 5.00 | .046 | -.630 |
| **SupCont2** | 1.00 | 5.00 | -.076 | -.581 |
| **SupCont1** | 1.00 | 5.00 | -.155 | -.963 |
| **ImpactT2** | 1.00 | 4.667 | -.801 | .695 |
| **SelfDT2** | 1.00 | 5.00 | -.733 | .238 |
| **CompT2** | 1.667 | 5.00 | -.758 | .261 |
| **MeanT2** | 2.00 | 5.00 | -.667 | -.176 |
| **TaskProf3T2** | 1.00 | 5.00 | .039 | -.853 |
| **TaskProf2T2** | 1.00 | 5.00 | -.204 | -.509 |
| **TaskProf1T2** | 1.00 | 5.00 | -.048 | -.752 |
| **TaskAdap3T2** | 2.00 | 5.00 | -.346 | -.271 |
| **TaskAdap2T2** | 1.00 | 5.00 | -.327 | -.032 |
| **TaskAdap1T2** | 2.00 | 5.00 | -.307 | -.384 |
| **TaskProa3T2** | 1.00 | 5.00 | -.664 | .581 |
| **TaskProa2T2** | 1.00 | 5.00 | -.670 | .252 |
| **TaskProa1T2** | 2.00 | 5.00 | -.385 | -.390 |
